# Objectively Monitoring Amyotrophic Lateral Sclerosis Patient Symptoms During Clinical Trials With Sensors: Observational Study

**DOI:** 10.2196/13433

**Published:** 2019-12-20

**Authors:** Luis Garcia-Gancedo, Madeline L Kelly, Arseniy Lavrov, Jim Parr, Rob Hart, Rachael Marsden, Martin R Turner, Kevin Talbot, Theresa Chiwera, Christopher E Shaw, Ammar Al-Chalabi

**Affiliations:** 1 Advanced Biostatistics & Data Analytics Centre of Excellence R&D Projects Clinical Platforms & Sciences GlaxoSmithKline Stevenage United Kingdom; 2 Translational Medicine Future Pipeline Discovery GlaxoSmithKline Stevenage United Kingdom; 3 Clinical Development AveXis Bannockburn, IL United States; 4 McLaren Technology Centre McLaren Applied Technologies Woking United Kingdom; 5 Nuffield Department of Clinical Neurosciences University of Oxford Oxford United Kingdom; 6 Maurice Wohl Clinical Neuroscience Institute Department of Basic and Clinical Neuroscience King’s College London London United Kingdom

**Keywords:** amyotrophic lateral sclerosis, objective symptom monitoring, clinical trial, physical activity, digital phenotyping, digital biomarker, heart rate, speech, accelerometer, wearable

## Abstract

**Background:**

Objective symptom monitoring of patients with Amyotrophic Lateral Sclerosis (ALS) has the potential to provide an important source of information to evaluate the impact of the disease on aspects of real-world functional capacity and activities of daily living in the home setting, providing useful objective outcome measures for clinical trials.

**Objective:**

This study aimed to investigate the feasibility of a novel digital platform for remote data collection of multiple symptoms—physical activity, heart rate variability (HRV), and digital speech characteristics—in 25 patients with ALS in an observational clinical trial setting to explore the impact of the devices on patients’ everyday life and to record tolerability related to the devices and study procedures over 48 weeks.

**Methods:**

In this exploratory, noncontrolled, nondrug study, patients attended a clinical site visit every 3 months to perform activity reference tasks while wearing a sensor, to conduct digital speech tests and for conventional ALS monitoring. In addition, patients wore the sensor in their daily life for approximately 3 days every month for the duration of the study.

**Results:**

The amount and quality of digital speech data captured at the clinical sites were as intended, and there were no significant issues. All the home monitoring sensor data available were propagated through the system and were received as expected. However, the amount and quality of physical activity home monitoring data were lower than anticipated. A total of 3 or more days (or partial days) of data were recorded for 65% of protocol time points, with no data collected for 24% of time points. At baseline, 24 of 25 patients provided data, reduced to 13 of 18 patients at Week 48. Lower-than-expected quality HRV data were obtained, likely because of poor contact between the sensor and the skin. In total, 6 of 25 patients had mild or moderate adverse events (AEs) in the skin and subcutaneous tissue disorders category because of skin irritation caused by the electrode patch. There were no reports of serious AEs or deaths. Most patients found the sensor comfortable, with no or minimal impact on daily activities.

**Conclusions:**

The platform can measure physical activity in patients with ALS in their home environment; patients used the equipment successfully, and it was generally well tolerated. The quantity of home monitoring physical activity data was lower than expected, although it was sufficient to allow investigation of novel physical activity end points. Good-quality in-clinic speech data were successfully captured for analysis. Future studies using objective patient monitoring approaches, combined with the most current technological advances, may be useful to elucidate novel digital biomarkers of disease progression.

## Introduction

### Background

Amyotrophic Lateral Sclerosis (ALS) is a neurodegenerative disorder affecting motor neurons, characterized by progressive weakness, leading to increased disability and eventually death from neuromuscular respiratory failure, typically within 5 years [1,2].

There is no known cure for ALS; the existing licensed disease-modifying medications riluzone (trade names: Rilutek, Teglutik), and edaravone (trade names: Radicut, Radicava) are only modestly effective in impacting the disease course or improving survival [3-5]. Thus, there remains a significant unmet medical need in ALS for therapies to slow progression of functional decline and improve survival. Numerous therapies and experimental agents have been tested in ALS and have failed, sometimes after positive results were achieved in early efficacy studies, suggesting a need to improve outcome measures of disease progression and overall study design [6,7]. Existing disease progression measures have limitations in terms of sensitivity, requiring long trials with large sample sizes. The ALS Functional Rating Score (Revised) (ALSFRS-R) [8] is the most commonly used instrument to monitor the progression of disability in patients with ALS; however, it relies on patients’ recollection of their clinical function rather than direct objective assessment. Testing burden in ALS clinical trials is heavy and often involves a significant number of in-clinic assessments for overall clinical function, respiratory function, and muscle strength, which are mainly conducted at clinical visits. This can be tiring for patients, particularly as the disease progresses, leading to patient dropout and, consequently, missing data. As a result, the ability to draw conclusions from the data is often impacted. In that regard, objective monitoring of patients has the potential to (1) enable the development of novel *digital* biomarkers to accurately quantify changes in function and disease progression with greater sensitivity than current clinical methods, thereby enabling smaller and shorter trials, and (2) provide additional, important information to assess the impact of the disease and treatment on clinical function and activities of daily living in a real-life setting, while reducing the testing burden.

For these reasons, there has been an increasing interest in developing new technologies for remote and objective clinical assessment of ALS, which may be useful outcome measures in clinical trials [9-11].

### Amyotrophic Lateral Sclerosis Digital Biomarker Candidates

Although the presentation of ALS varies among patients, its main characteristics are (1) upper and lower motor neuron symptoms and signs, resulting in skeletal muscle weakness and spasticity (compromising mobility and activities of daily living), (2) speech and swallowing difficulties, and (3) respiratory problems. In addition, other symptoms such as impaired cardiac autonomic control, weight loss, cramps and fasciculations, emotional lability, and frontal lobe-type cognitive dysfunction are not uncommon [10,12-16]. Technology advances over the last few years have provided an opportunity to objectively quantify some of these manifestations.

Movement sensors have been previously used to quantify mobility and activities of daily living in patients with rheumatoid arthritis (RA), chronic obstructive pulmonary disease (COPD), or Parkinson disease (PD). Hashimoto et al [17] found that the mean daily activity level of patients with RA was significantly lower than that in healthy controls, and the number of sedentary periods was significantly higher and moderately correlated with the Health Assessment Questionnaire Disability Index. Van Buul et al [18] found that patients with more symptomatic COPD do fewer steps a day and spend less time in moderate-and-vigorous physical activity than those with less symptomatic disease. Lipsmeier et al [19] found multiple aspects of significantly reduced everyday motor behavior in patients with PD compared with controls. Objective activity data have not been reported for patients with ALS thus far, although ongoing studies (such as the AT HOME study [9]) aim to investigate the use of armbands for evaluating disease progression through physical activity measures. Patients are anticipated to be increasingly less mobile as the disease progresses, which would be reflected in multiple activity measures, including overall time spent active, average daily activity levels, or activity fragmentation. In this study, we hypothesized that these measures could be objectively assessed with a standard accelerometer, as long as patients wear the device for a period of time long enough to be representative of their typical behavior (continuous in-home monitoring).

Portable electrocardiogram (ECG) systems have been used to evaluate cardiac function and perform analysis of heart rate variability (HRV) data. An increase in the mean heart rate at rest, a decrease in standard deviation of interbeat interval (tRR), as well as in proportion of number of pairs of successive beat-to-beat intervals that differ more than 50 ms divided by the total number of beat-to-beat intervals, and an increase in the low-frequency/high-frequency (LF/HF) component ratio were found in patients with ALS, indicating a vagal-sympathetic imbalance [10,20,21].

Different acoustic recording systems have previously been used to demonstrate associations between acoustic measures and speech intelligibility in patients with ALS. Patients were found to have significantly slower syllabic and speaking rate and lower speech intelligibility, with speaking rate typically declining much earlier in the disease stage than speech intelligibility [11,22,23]. In this study, we evaluate speech characteristics at the clinical sites to allow for high-fidelity data capture equipment and a controlled quiet environment that may allow more accurate quantification of changes in speech formats.

All previously reported efforts to objectively monitor patients with ALS evaluate individual symptoms only; however, the heterogeneity of symptom presentation (among patients and within a patient over time) makes it likely that a combination of measurements would be needed to accurately quantify changes in disease progression with high sensitivity for a diverse cohort of patients. Thus, platforms incorporating multiple objective symptom assessment technologies present the best opportunity to investigate multiple digital biomarkers—and ultimately composite digital biomarkers—that would be useful measures of disease progression for a broad patient population and disease stages.

The objective of this clinical study was 3-fold: (1) to investigate the feasibility of a novel platform for objective data collection of multiple ALS manifestations (physical activity, HRV, and speech characteristics) in a clinical study; (2) to explore the impact of the devices on patients’ everyday life and to record safety and tolerability related to the devices and study procedures over 48 weeks; and (3) to explore digital disease progression markers in ALS, which may be useful outcome measures in clinical trials. The results for the latter objective will be reported separately.

## Methods

### Study Overview

**Figure 1:**
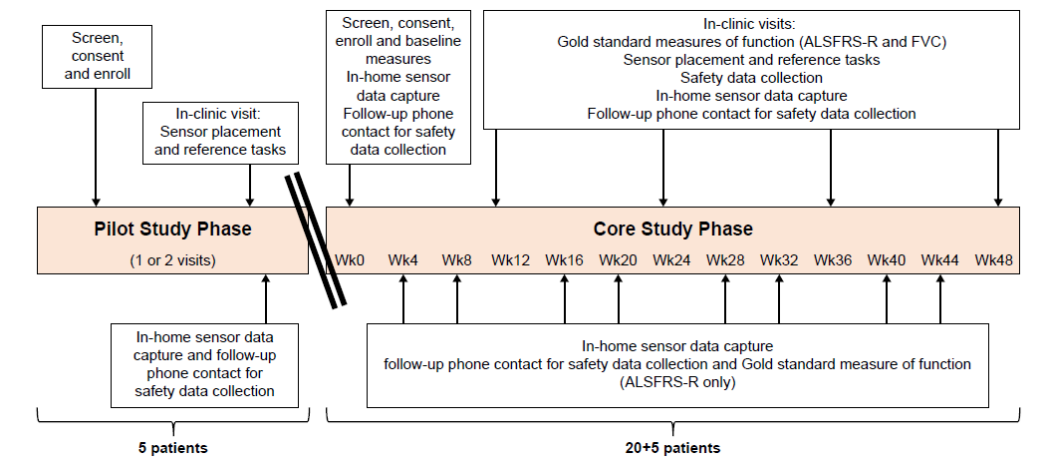
Study design overview: All 5 patients from the Pilot Study Phase progressed to the Core Study Phase. ALSFRS-R: ALS Functional Rating Score (Revised); FVC: forced vital capacity; Wk: week.

An exploratory, noncontrolled, nondrug study in ALS (NCT02447952) was sponsored by GlaxoSmithKline (GSK) and conducted in collaboration with McLaren Applied Technologies (MAT). A total of 2 clinical sites, both in the United Kingdom, enrolled patients. The study was initiated on June 30, 2015 (first patient screened), and completed on June 1, 2017 (last patient, last visit). The study comprised 2 phases, a variable length Pilot Study Phase (5 patients enrolled) and a 48-week Core Study Phase (25 patients enrolled, including the 5 patients who progressed from Pilot Study Phase to Core Study Phase), as shown schematically in Figure 1. The 2-phase approach was utilized to allow for refinement of the equipment and data transmission processes during the Pilot Study Phase and enable adaptation of components of the digital platform, which were performing suboptimally before embarking on the Core Study Phase. In such an exploratory setting, the 2-part design helped mitigate taking forward equipment, algorithms, data capture methods, and data transmission processes that may require adaptation and lessened the risk that major changes would be needed during the Core Study Phase. For the duration of the study, patients attended a clinical site visit every 12 weeks to perform various assessments and tasks; in addition, they wore a sensor in their daily life for approximately 3 consecutive days every month (home monitoring periods), allocating 2 hours per 24-shour period to recharge the sensor. The study did not include any specific patient retention strategy. The home monitoring period duration was selected to minimize patient burden while capturing sufficient data to enable extraction of clinically relevant data. At all study time points, patients were provided with a diary as a tool to record details about their experience with the sensor and their activity type and level while wearing the sensor.

The study was designed to enable exploration of novel disease progression markers; for the purposes of the sample size justification, the correlation between the ALSFRS-R and the novel end points was considered. With 20 patients, it was assumed that there would be an 80% chance of detecting a within-patient correlation of greater than or equal to 0.6 (considered a moderate to strong correlation), if the true correlation was 0.7. A total of 25 patients were enrolled to allow 20 evaluable patients (anticipating 20% dropout rate).

### Patient Population

The intended study population comprised patients with a diagnosis of ALS, who were ambulant and had a relatively high level of clinical function at baseline. The diagnosis was required to have been made by a neurologist with ALS expertise within 18 months of symptom onset. Eligible participants were 18 to 80 years of age, capable of giving signed (or verbal) informed consent and were capable of, and willing to follow the study protocol. Patients were excluded from study participation if they met any of the following criteria: had neurological (other than ALS) or nonneurological comorbidities; presented with clinically significant cognitive impairment; had a regionally restricted form of ALS or other atypical variant; required mechanical ventilation; and had an active implantable cardiac medical device or were at a high risk for needing external defibrillation or had a history of skin hypersensitivity to adhesives. There were no prohibited medications, but enrollment into an investigational drug trial (in addition to participation in this trial) could be prohibited if, in the opinion of the investigator, the investigational drug might have impacted the objectives of this study. Table 1 summarizes the baseline population characteristics of all patients and the completers’ population. As expected, the most common concomitant medications were from the nervous system class, with the most common being riluzole (64%), zopiclone (24%), and citalopram (20%; Table 2).

**Table 1:** Patient demographics and baseline disease characteristics.

Characteristics		Overall population (N=25)	Completers population (n=18)
**Gender, n (%)**			
	Male	21 (84)	16 (89)
	Female	4 (16)	2 (11)
Age (years), mean (SD)		53.1 (9.93)	52.9 (11.3)
**Race, n (%)**			
	White	23 (92)	17 (94)
	Asian	2 (8)	1 (6)
**Duration of Amyotrophic Lateral Sclerosis, n (%)**			
	<18 months	22 (88)	16 (89)
	Missing	3 (12)	2 (11)
**Phenotype at onset, n (%)**			
	Upper limbs	15 (60)	10 (56)
	Lower limbs	6 (24)	5 (28)
	Upper and lower limbs	2 (8)	1 (6)
	Bulbar	2 (8)	2 (11)
ALSFRS-R^a^ total score, mean (SD)		41.6 (4.98)	43.0 (2.71)
FVC^b^, mean (SD)		3.927 (1.432)	4.262 (1.238)

^a^ALSFRS-R: Amyotrophic Lateral Sclerosis Functional Rating Score (Revised).

^b^FVC: forced vital capacity.

**Table 2:** Most common (≥2 patients) concomitant medications.

Anatomical Therapeutic Chemical medication grouping		Core Study Phase (N=25), n (%)
**Any medication**		22 (88)
	Nervous system	20 (80)
	Musculoskeletal system	4 (16)
	Alimentary tract and metabolism	3 (12)
	Cardiovascular system	3 (12)
	Sensory organs	3 (12)
	Genitourinary system and sex hormones	2 (8)
	Respiratory system	2 (8)

### Technology Description

**Figure 2:**
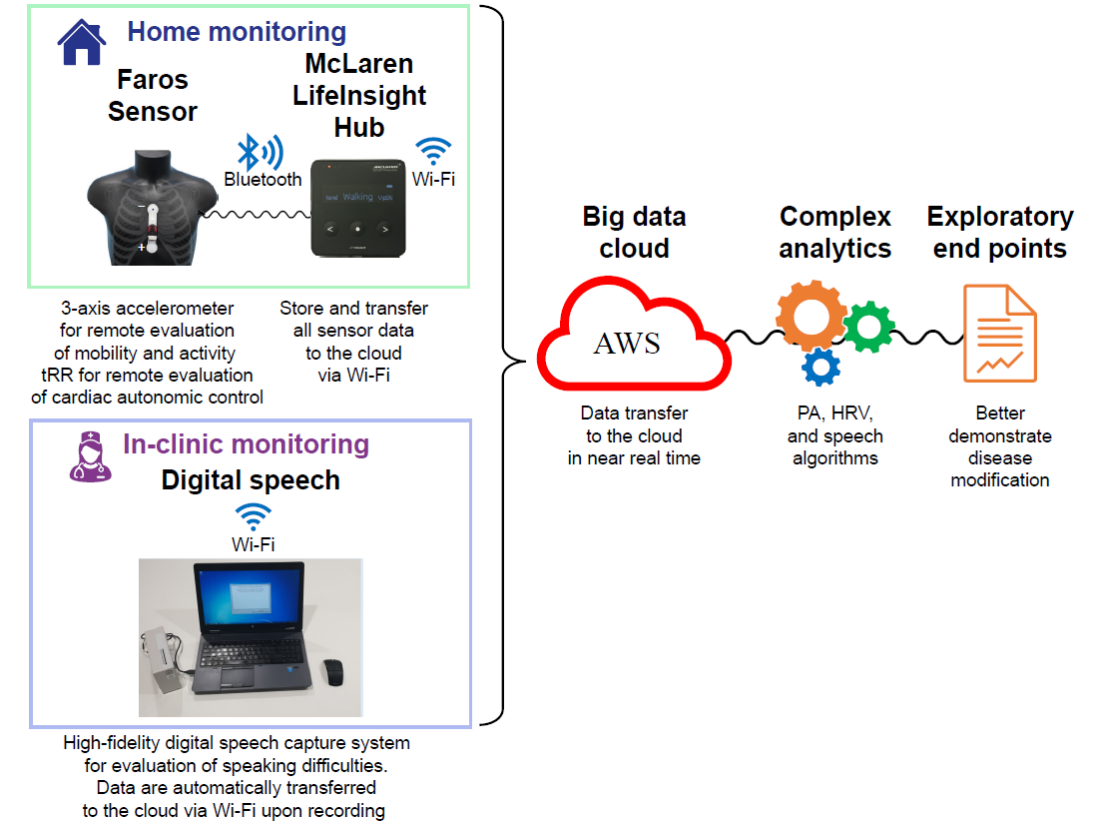
Schematic representation of the monitoring platform. AWS: Amazon Web Service; HRV: heart rate variability; PA: physical activity; tRR: interbeat interval.

A monitoring system (Figure 2) was developed to allow evaluation of key ALS symptoms. It comprised the following 3 main components:

1. The commercially available Mega Faros 180 accelerometer (3 axes, 50 Hz) and 2-lead ECG sensor (Mega Electronics Ltd, Finland). The selected heartbeat sensing electrode was the Mega Fast Fix electrode disposable patch. The accelerometer and the electrode were attached to the chest (Figure 2). It was expected that physical activity and tRR data would be continuously captured by this sensor (extracted from 1 kHz ECG data, enough for HRV analysis [24]; full ECG data are not available with the sensor configuration utilized). This device is in conformity with the provisions of the Council Directive 93/42/EEC of June 14, 1993, concerning medical devices, and it was compliant with the standards of measurement from the European Society of Cardiology and the North American Society of Pacing and Electrophysiology [25].
2. A “LifeInsight Hub (v.2.0.6.),” developed by MAT, received data from the sensor via a secure Bluetooth wireless signal every 2 min. The hub then automatically uploaded the data in near real time (every 10 min) to secure cloud servers (Amazon Web Services EC2) via a secure connection on a third-generation mobile phone network. The hub allowed secure connection to additional devices, and it may be utilized to receive and transmit data from different data capture technologies, depending on future study needs.
3. A digital speech capture system comprising a high-fidelity microphone connected to a computer, with bespoke software that instructed the patients to say a series of vowels, words, and paragraphs, which were then recorded and immediately automatically transferred to a secure server via mobile connectivity.

### Physical Activity

A total of 2 types of activity algorithms (summarized in the Multimedia Appendix 1) were developed to interpret the raw data from the accelerometer: “Activity score” algorithms to evaluate “how much” activity the patients performed and “Activity classification” algorithms to evaluate “what” activities the patients performed.

The activity score is used as a measure of physical activity and is based on the intensity of movement. To develop the activity classification algorithms, a series of reference tasks were performed by the patients at each clinic visit in both the Pilot and Core Study Phases. A group of healthy volunteers outside the clinical study also performed the same tasks before the Core Study Phase; this can enhance algorithm accuracy when the number of patients is small [26]. All the reference tasks served as a “blueprint” for specific movements measured by the accelerometer. The data generated from these tasks helped in developing the algorithms and evaluating their performance using a leave-one-patient-out validation procedure. The reference tasks (which included sitting, standing, lying down, walking, climbing stairs, and transitions such as sit to stand, stand to sit, stand to lying, lying to stand, and nine-hole peg test of manual dexterity) were predefined to enable the development of clinically meaningful digital markers of disease progression for investigation. This process aligns with the recommendations from the Clinical Trials Transformation Initiative [27].

### Heart Rate Variability

There are a number of widely accepted HRV metrics [17]; 2 such metrics include the Root Mean Square of the Successive Differences (RMSSD; time domain) and LF/HF ratio (frequency domain).

It is important to note that measures of HRV are derived from tRR data and are impacted by the duration of the time series (number of data points), body orientation, time of day, and activity being performed. Where possible, these factors have been considered by using the recommended 5-min duration of tRR and providing values specific to each activity.

From our analysis (summarized in the Multimedia Appendix 1), the RMSSD metric is less sensitive to the amount of data points missing. As per our analysis (Multimedia Appendix 1), around 88% of the data was needed to reliably compute RMSSD for a 5-min window, whereas almost all the data (99%) were needed to reliably compute LF/HF. Therefore, the RMSSD may be considered a metric that is more robust for patients with lower data quality. This is a similar finding to other studies that have found time-based metrics to be less sensitive to missing data and poor data quality than frequency-based metrics [28].

### Speech

The speech data collection was performed during the clinical site visits for simplicity and to allow for a more sensitive and structured assessment at this early exploratory stage. Speech algorithms to extract acoustic, quantitative, and linguistic audio features were developed based on classical speech processing techniques (eg, formants and fundamental frequencies) and used, where possible, open-source components to implement these techniques.

The patients were asked to perform 4 speech tests that were identified as clinically relevant. The first 2 tests used the phonation of the “Ah” sound, one being shortly sustained and repeated 7 times and the other being a long-sustained sound for 10 seconds [22]. The subsequent test was to pronounce the word “doily” 3 times [11]. Finally, the patients were asked to read a short 100-word paragraph (“Bamboo” passage) [29]. This particular speech data collection protocol comprised tests that had individually been shown to demonstrate associations between acoustic measures and speech intelligibility in patients with ALS but who had not been jointly investigated within the same patient group.

In contrast to physical activity and HRV, the data and processing of speech were done offline using a MATLAB script to analyze the speech data and generate a comma-separated value file with the desired end points. This process is summarized in the Multimedia Appendix 1.

## Results

### Pilot Study Phase: Feasibility of Equipment and Data Transmission

A total of 5 patients with ALS were recruited for the Pilot Study Phase and attended 2 clinical visits. Patients also wore the sensor in their routine home-life setting for approximately 3 days after each clinical visit.

At the time of reviewing the data from the Pilot Study Phase, clinic reference task data were available for 4 of the 5 patients and home monitoring was available for 3 out of 5 patients. The missing data did not propagate through the system within the expected timeframe, but data were subsequently fully recovered. For every patient, the number of days having least 18 hours’ data available varied from 2 to 3. The device recharging routine (2 hours each day) was successfully adopted by 3 of the 5 patients.

HRV data were available for 4 of the 5 patients and the percentage of success of good-quality HRV windows of data varied from 74.8% to 100% for RMSSD analyses and 53.2% to 100% for LF/HF analyses. This was deemed to be acceptable and no adaptations for the Core Study Phase were considered necessary. The amount and quality of the speech data captured were as planned.

Most (4; 80%) patients were able to attach and remove the sensor without assistance. A patient reported that the sensor fell off on a single occasion. Most patients (4; 80%) found the device comfortable to wear. All patients reported that wearing the sensor did not have an impact on the ability to perform daily activities. Overall, the sensor was well tolerated, although 1 of the 5 patients experienced mild skin irritation.

Satisfactory user acceptance for the new technology, combined with the successful capture of study data and absence of adverse events (AEs), enabled the study to continue to the Core Study Phase with minimal tweaks to the monitoring platform. No protocol amendments based on the Pilot Phase results were necessary.

### Core Study Phase

A total of 25 patients (including the 5 patients who completed the Pilot Study Phase) were enrolled in the Core Study Phase. A total of 18 of the 25 patients completed the study; 4 patients discontinued from the study because of withdrawal of consent (3 of whom had become too unwell to continue with the study because of progression of ALS, the fourth patient did not provide a specific reason), 2 patients discontinued because of AEs, and 1 patient was discontinued at the investigator’s discretion. The mean (standard deviation) ALSFRS-R score at baseline was 41.6 (4.98). The mean (standard error) monthly rate of change for ALSFRS-R total score was 0.9 (0.23) points/month.

#### Physical Activity and Heart Rate Variability: Impact, Data Quantity, Quality, and Algorithm Accuracy

At the start of the Core Phase, a majority of patients (16/25, 64% patients) were able to attach and remove the sensor without assistance; however, the proportion of patients who did not require assistance gradually decreased over the course of the study, and by Week 48, only 6 of 15 patients (40.0%) were able to attach the sensor without some assistance. A majority of patients found the device comfortable to wear; the majority of patients who reported that the device was uncomfortable reported symptoms of skin irritation (skin itching and local skin reactions likely because of an allergy to the adhesive). A majority of patients reported that wearing the sensor had no or minimal impact on the ability to perform daily activities during the Core Phase; only 1 patient reported a moderate impact on daily activities at Week 12.

A low number of patients reported a minimal impact of the sensor on sleep: the reasons given included difficulty in getting comfortable, local itching, and impact of the flashing light at night. A patient reported a moderate negative impact on sleep.

Most patients adhered to protocol requirements on sensor wear: 65% of visits with data across the subjects had at least three days of data. However, only 6 of 25 (24%) patients had data captured for all 13 home monitoring time points.

**Figure 3:**
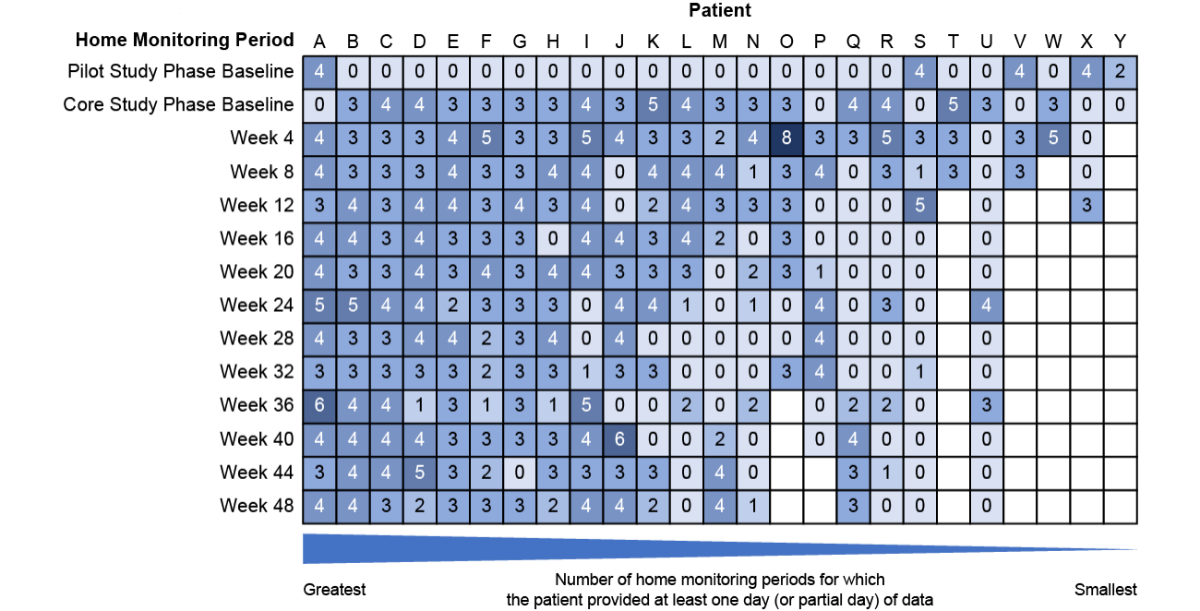
Home monitoring data coverage: number of days (or partial days) of data captured for each patient and time point. Blank entries indicate early withdrawals. Patients had either a Pilot Study Phase or Core Study Phase baseline home monitoring period but not both. For clarity, in 5 instances, data were excluded from the analysis because of predefined data quality deviation rules unrelated to the monitoring platform.

Figure 3 illustrates the number of days of raw accelerometer and tRR data captured for each patient at each time point. There are examples of good data coverage where the patient has been able to follow the protocol (eg, patients A, B, and C) and many examples of deviations from the ideal data coverage. Patients O, P, T, V, W, X, and Y ended the study early.

There are significant gaps in the data coverage for patients Q, R, S, and U, as indicated by the zeros in Figure 3. Others have smaller gaps in the data coverage. Across all 25 patients, no data were collected for 24% of time points expected.

There are several instances where more than 3 days, or partial days, of data were collected (partial days represent those days where any data were captured). Patient O used the sensor for 8 days during 1 home monitoring period, and there are several occurrences of 5 or 6 days of wear time across different patients.

**Figure 4:**
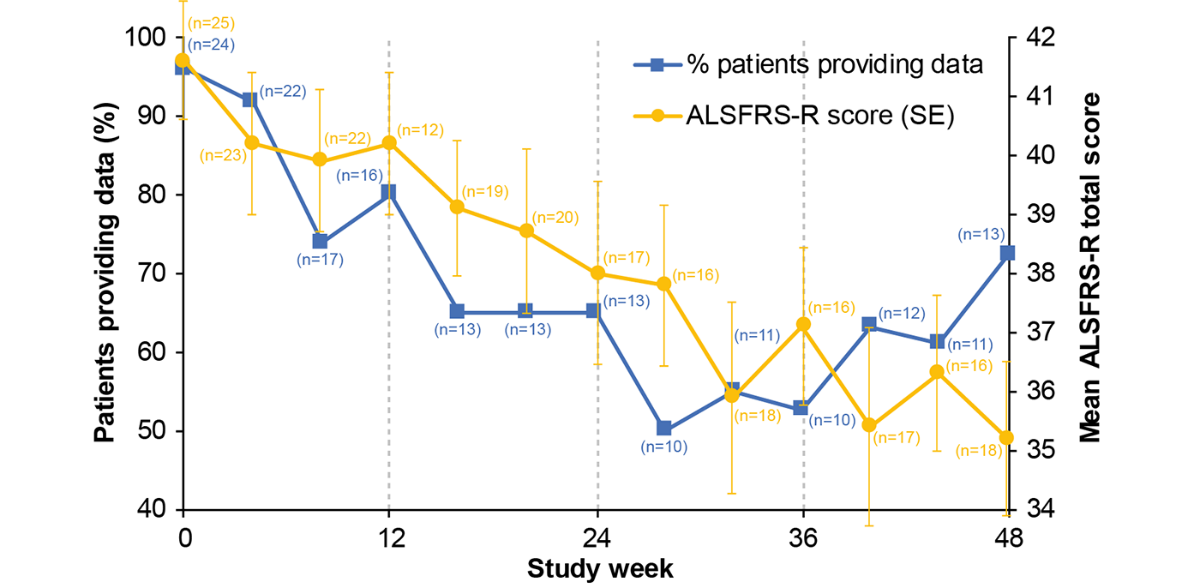
Percentage of patients providing home monitoring data throughout the study; mean Amyotrophic Lateral Sclerosis Functional Rating Score (Revised) total score of all patients remaining in the study. The number in brackets next to each time point represents the number of patients in the study. ALSFRS-R: ALS Functional Rating Score (Revised).

At baseline, 23 of 25 (92%) patients provided data for 3 or more days (or partial days) during the home monitoring; however, at Week 48, this reduced to 10 of 18 (56%) patients, indicating that either an increasing number of patients were unable to meet the protocol requirements as the study progressed or they were less willing to comply with study procedures. Over the course of the study, there was deterioration in the ALSFRS-R total score, indicating worsening of ALS (loss of physical function), and a decreasing number of patients provided any home monitoring sensor data (Figure 4).

If a patient wore the sensor for any period during the day which was significantly lower than the requested 22-hour period, then the derivation of any activity type end point, even if normalized to wear time, may be biased because of the heterogeneity of activity patterns throughout the day. The total wear times over each 24-hour period showed that patients did not wear the device for long enough. Average total wear time over the 24-hour recording period showed mean total wear time was 1106 min (approximately 18.4 hours) per day at baseline. The mean total wear times did not decrease significantly throughout the study to Week 48 (1084 min; 18.1 hours) per day and did not decrease significantly from the first day of monitoring, “Day 1” (1086 min; 18.1 hours) to the third, “Day 3” (1051 min; 17.5 hours). Therefore, although fewer patients provided data as the study progressed, the quality of the daily data provided by participating patients did not deteriorate significantly.

**Figure 5:**
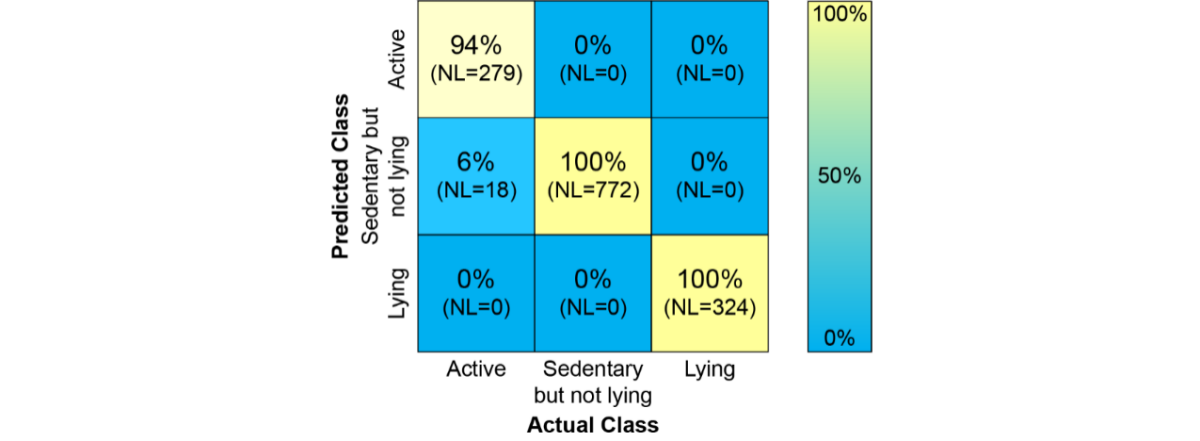
The confusion matrix for the physical activity algorithms. The percentages represent the predictions of “Actual classes.” NL: number of data labels from reference tasks, collected by 24 Amyotrophic Lateral Sclerosis patients (1 patient did not provide reference task data). Each label contains 1 min of accelerometer data.

Physical activity algorithms were developed to classify “active,” “sedentary but not lying,” and “lying,” as these 3 activity classes were expected to enable describing patients’ level of physical function. For clarity, “sedentary but not lying” refers to any activity requiring low levels of intensity (but excluding “lying down”), such as standing, sitting, or performing low-intensity movements. The algorithms were tested using the reference tasks data. Their overall performance is illustrated by the confusion matrix in Figure 5, where it can be seen that a few “active” labels are being predicted as “sedentary but not lying”; nonetheless, the overall performance is considered high, as accuracy, sensitivity, and specificity are greater than 97% for all 3 activity classes.

Overall, the amount and quality of the data collected and the accuracy of the algorithms developed were sufficient to evaluate changes over time in patients’ activities of daily living. Changes from baseline at Week 48 in the main physical activity end points investigated (normalized to wear time) are shown in Table 3. A reduction in the patients’ ability to perform activities of daily living over time can be observed across all end points.

**Figure 6:**
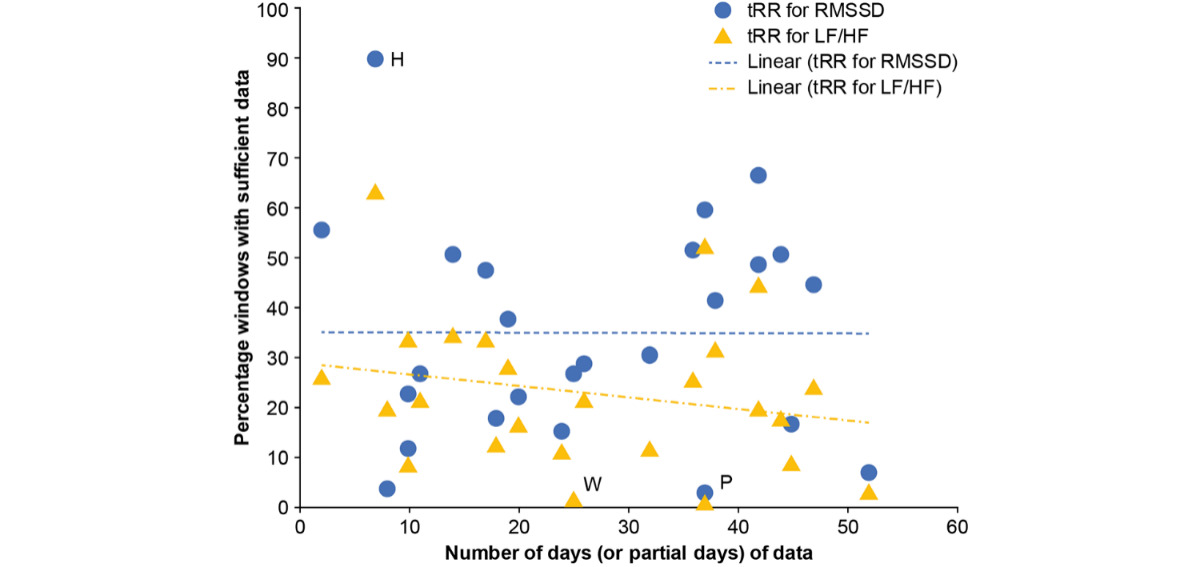
Percentage of windows with sufficient data to compute heart rate variability metrics for each patient. LF/HF: low-frequency/high-frequency; RMSSD: Root Mean Square of the Successive Differences; tRR: interbeat interval.

Figure 6 provides a high-level summary of the HRV data quality for each patient across the study. The effect of muscle activation can be considered small enough, which was supported by the tRR data. Some patients had poor data quality throughout the study, whereas others had periods of both good and poor data. Very few patients had good data quality throughout the study. The percentage of windows with enough good-quality data to compute the RMSSD and LF/HF metrics varied across the patients. Some patients had very poor data quality, with very few windows having sufficient data to compute the HRV metrics (eg, patient P and patient W). It is possible that those patients had a poor contact between the sensor and the skin using the Fast Fix.

**Table 3:** Changes in physical activity end points from baseline to Week 48.

End point		Baseline (n=24), mean (SE)	Week 48 (n=13), mean (SE)
Average daytime active (minutes)		34.42 (6.00)	23.58 (8.05)
Percentage of daytime active (%)		5.36 (0.85)	3.59 (1.16)
Average daytime sedentary (minutes)		602.22 (26.89)	651.11 (43.36)
Percentage of daytime sedentary (%)		94.64 (0.85)	96.41 (1.16)
Total daytime activity score per hour (counts)		3336.55 (541.83)	1972.51 (612.89)
Total 24-hour activity score per hour (counts)		2275.65 (370.22)	1430.35 (464.89)
Maximum daytime activity score per hour (counts)		2803.91 (575.50)	1647.04 (456.40)
Mean maximum daytime activity score per hour (counts)		1618.40 (263.01)	1002.06 (280.53)
**Daytime number of active periods per hour (minutes)**			
	>1 to ≤2	0.28 (0.06)	0.25 (0.10)
	>2 to ≤5	0.11 (0.03)	0.10 (0.04)
	>5 to ≤15	0.04 (0.02)	0.02 (0.01)
Average duration of active periods >1 min (minutes)		2.60 (0.24)^a^	2.08 (0.13)^b^

^a^n=21.

^b^n=7.

Some patients had much better data quality. In particular, nearly 90% of the windows for patient H had sufficient data to compute RMSSD; however, this patient only wore the sensor for 2 home monitoring periods (7 days of data in total); therefore, this high percentage is biased by the limited wear time (Figure 3).

For all data across the whole study, only 33% of 5-min windows of tRR data were sufficient to compute RMSSD metrics, and only 19% were sufficient to compute the LF/HF metrics. Overall, the percentage of RMSSD and LF/HF quality data did not correlate with the number of days of data; there was only a slight decrease in LF/HF quality data, with increasing number of days of data.

A slight decrease in the RMSSD HRV mean and variance data was observed over time (data not shown), but this finding should be treated with caution, given the small amount of data available. Unsurprisingly (because of the limited amount of LF/HF data available), no discernible trends were observed over time in the LF/HF data.

#### Speech: Data Quantity and Quality

The quantity and quality of the digital speech data files captured during the Core Study Phase of the study was as intended, with no significant issues related to the methods or equipment. Both at baseline and at Week 48, all (100%) patients captured the digital speech data successfully. However, patients J, P, and U did not perform the speech test at Weeks 12, 24, or 36. All data files captured were transferred as anticipated, and no data were lost. All speech data were successfully analyzed. Change from baseline values at Week 48 were low for all speech end points studied, with no obvious pattern of change over time.

#### Safety Evaluation: Adverse Events and Serious Adverse Events

Safety evaluation comprised reported AEs and serious AEs (SAEs) related to the study equipment, devices, or procedures only. A total of 6 (24.0%) patients reported AEs assessed as related to the devices (including the sensor and Fast Fix adhesive patches); there were no reports of SAEs and no deaths, and only 2 (8.0%) patients had AEs that led to withdrawal, both because of contact dermatitis. Table 4 summarizes AEs in the study.

All the AEs reported in the study were in the skin and subcutaneous tissue disorders system organ class (SOC) and all were skin reactions related to the use of the Fast Fix adhesive patch. The most commonly reported AE was contact dermatitis.

A total of 21 AEs were reported by 6 patients. Events were mild or moderate in intensity in 5 of 6 (83.3%) patients and all events were recovered/resolved at the end of study (1 patient had an AE of contact dermatitis, which was recovered/resolved with sequelae). A patient had 2 events of pruritic rash of severe intensity: the first event lasted for 4 days; the second event lasted for 5 days.

**Table 4:** Summary of adverse events in the Core Study Phase (N=25).

Adverse event		Adverse events reported	Patients reporting adverse events
Adverse events		21	C, F, J, U, W, and Y
**Severity of events**			
	Mild	14	Y, F, J, and C
	Moderate	5	Y, U, and W
	Severe	2	U
	Severe adverse events	0	—^a^
**System organ class**			
	Skin and subcutaneous tissue	21	C, F, J, U, W, and Y
**Disorders**			
	Dermatitis contact	15	Y, F, U, and W
	Rash	3	J and C
	Rash pruritic	2	U
	Skin irritation	1	Y
Adverse events leading to withdrawal		2	W and Y
Deaths		0	—

^a^No patient reported an adverse event.

#### Medical Device Incidents, Near-Miss Incidents, and Malfunctions

A near-miss incident was reported in the Core Study Phase: it was discovered that, despite being CE-marked, in some circumstances, it was possible for the mains chargers’ casing to come apart, exposing the internal wires and components, resulting in a risk of an electrical shock. The clinical sites were informed immediately, and all the chargers were recalled from all the patients and replaced. A patient reported a replacement charger made crackling noises and smelled of burning when it was plugged into the mains. The issue was reported to the manufacturer, who confirmed there were no other instances of failure in over 15,000 units sold over a 5-year period before receiving this report. The patient was issued with a replacement charger. Before receiving the new charger, the patient had been able to use a mobile phone charger; therefore, no data were lost.

No incidents or malfunctions were reported with the use of Mega Faros sensor, Fast Fix electrode, or LifeInsight hub in this study.

## Discussion

### Principal Findings

Objective monitoring of disease manifestations in clinical trials using sensors is a rapidly advancing area, with over 100 actively recruiting trials using technology-based outcomes across a range of diseases (as of July 2019), according to clinical trial registers [30]. Interest is building across multiple conditions, including other neurodegenerative diseases, such as PD and multiple sclerosis [31]; RA [26,32], and COPD [33]. This study successfully developed and explored the feasibility of a novel integrated platform to objectively monitor multiple ALS symptoms across several domains (physical activity, HRV, and speech). This is a key advantage compared with other existing approaches that monitor symptoms individually [10,34]. Moreover, the platform is flexible and could be tailored to the needs of future studies, as components for measuring different symptoms may be added or removed as required. The system allowed for real-world and in-clinic data collection, both of which could be reviewed remotely in near real time.

The dual-phase study design proved to be useful, as the Pilot Study Phase enabled adaptation of components and algorithms before embarking on the Core Study Phase, thereby lessening the risk that major changes would be needed during the Core Study Phase. From an ethical perspective, this is particularly important in ALS, as patients have a limited life expectancy and are making a significant effort donating their time to the study; therefore, clinical trial sponsors have a duty to maximize the value of their time and data collected. It is also important to note that the study participants continued to receive ALS standard of care (eg, riluzole and multidisciplinary care).

Core Study Phase results demonstrate that the monitoring platform can measure physical activity and digital speech for patients with ALS over the course of the 48-week study, although the specific sensor and analysis methodology used were not successful for continuously measuring tRR data, and, hence, HRV.

Importantly, this novel study showed that it was possible to assess the physical activity of patients with ALS in their daily life (real-world monitoring): the data propagated through the system and were received as expected, although in some instances, the data experienced delay in being transmitted because of poor network connectivity, which prevented the data to be available for review in true real time. At baseline, 23 of 25 patients collected at least three days of home monitoring data; however, this decreased to 10 of 18 patients at Week 48. The decreasing amount of data collected with study progression may be partly explained because of loss of physical function with disease progression; patients had increased reliance on carers to attach or remove the device and were less able to follow the study procedures. It might also be possible that patients were simply willing to provide less data as the “novelty” effect faded away over time. This highlights that it is important to achieve a balance between patient burden and the ease of use vs the amount of the data being collected. If technology is too intrusive, awkward, or requires frequent interaction with patients, then it is likely that compliance will be adversely affected, reducing the amount of valuable and useful data collected. Wrist-worn devices have shown excellent patient acceptance in previous studies [35]; however, they are currently unable to monitor HRV continuously, and from a biomechanical perspective, the wrist is a less-than-ideal wear location for accurate activity classification.

HRV data quality appeared to be affected by a poor connection between the Fast Fix electrode patch and the chest, yielding insufficient HRV data on which one may draw any robust clinical conclusions for both the RMSSD and LF/HF analyses. The lower than anticipated quality of HRV data could have been prevented if we had identified this as an issue at the end of the Pilot Study Phase. However, the Pilot Study Phase data showed a much greater percentage of good-quality HRV windows; this was likely biased because of the low number of patients and monitoring periods. A greater number of patients and/or monitoring periods would have been needed at the Pilot Study Phase to identify this issue. In future studies, data quality could be improved by optimizing study design and using newer, less invasive sensors with a more reliable skin attachment and not needing to be recharged daily. Owing to the rapid technological evolution that has occurred in the wearable device market, wearable devices are now available and are expected to continue improving reliability of data collection over time, but they were not available at the time this study was designed. Moreover, the analysis of HRV data in this study was performed using the typical 5-min duration of tRRs, but the literature is unclear as to how long HRV analysis windows should be to offer the best compromise between the quality/accuracy of the metrics extracted and the quantity of data resulting from the analysis. The longer the analysis window, the more reliable the HRV parameters; the shorter the analysis window, the lower the amount of data that needs to be excluded from analysis because of data points missing. Recent studies suggest that 2-min windows and 3-to-4 min windows would not negatively affect the quality of RMSSD and LF/HF analyses, respectively [36]; therefore, these might be a better choice to maximize data availability.

The amount and quality of the speech data captured were as intended, and there were no significant issues with the methods, equipment, or the data analysis. However, speech analysis was performed only during in-clinic visits. Future studies may employ different technologies, such as mobile phones, to collect digital speech data at home and more frequently. This approach is currently being followed in a number of ongoing studies, such as the AT Home study in ALS [9] or the mPower study in PD [37]. From a statistical perspective, more frequent data collected at home would be useful to investigate novel end points, particularly if within-patient and between-patient end point variability is high.

Patients were able to use all the study equipment successfully, with the majority of patients reporting that the sensor was comfortable to wear and had no or minimal impact on their ability to perform daily activities, nor did it affect their sleep quality. The technology was generally well tolerated with AEs in the skin and subcutaneous tissue disorders SOC reported by 6 of 25 (24.0%) patients, with the events being mild or moderate in intensity in 5 of 6 (83.3%) patients. This is a typical drawback of using sensors that are directly affixed to the skin; wearing armbands or wrist-worn devices often lessens the risk of skin irritation, but these sensors are less appropriate for continuous HRV monitoring.

The near-miss incident with the study technology, which occurred during the study, highlighted the importance of patient safety considerations that need to be taken into account before deciding upon which technologies are acceptable to use. Thorough risk assessments need to be carried out and risk management measures put in place before study starts, in addition to the use of CE-marked (or equivalent) equipment.

### Conclusions

In conclusion, the novel monitoring platform tested in this exploratory study was successful in collecting ALS patient data remotely, which may be useful in identifying digital markers of disease progression; the home monitoring of physical activity was a particular accomplishment that may generate insights into the real lives of patients with ALS not previously appreciated, thus providing the clinicians with a valuable new understanding of their patients’ well-being. The limitations of the study included a large amount of missing data, partly because of a number of patients withdrawing from the study early, which limited the ability to draw robust clinical conclusions; however, the platform was able to capture objective data to monitor patients outside of clinical visits, in near real time, and further improvements are expected with the ever-rapid advancements in digital sensor technologies. The relationship between the exploratory biotelemetry end points and the clinical gold standard ALS measures will be reported separately (manuscript under preparation). Ultimately, the goal should be to develop a platform that seamlessly integrates the data from various sources and standardizes ALS clinically relevant measures, procedures, and data reporting and validation and, ultimately, regulatory acceptance of the digital clinical trial end points.

## Appendix

Multimedia Appendix 1 Additional methodological information on the algorithms used for physical activity, heart rate variability, and speech.

## Abbreviations

AE: adverse event
ALS: Amyotrophic Lateral Sclerosis
ALSFRS-R: ALS Functional Rating Score (Revised)
COPD: chronic obstructive pulmonary disease
ECG: electrocardiogram
GSK: GlaxoSmithKline
HRV: heart rate variability
LF/HF: low-frequency/high-frequency
MAT: McLaren Applied Technologies
PD: Parkinson disease
RA: rheumatoid arthritis
RMSSD: Root Mean Square of the Successive Differences
SAE: serious adverse event
SOC: system organ class
tRR: interbeat interval

## References

[1] Kiernan MC, Vucic S, Cheah BC, Turner MR, Eisen A, Hardiman O, Burrell JR, Zoing MC (2011). Amyotrophic lateral sclerosis. Lancet.

[2] Talbot K (2009). Motor neuron disease: the bare essentials. Pract Neurol.

[3] Bensimon G, Lacomblez L, Meininger V (1994). A controlled trial of riluzole in amyotrophic lateral sclerosis. ALS/Riluzole Study Group. N Engl J Med.

[4] Fang T, Al Khleifat A, Meurgey J, Jones A, Leigh PN, Bensimon G, Al-Chalabi A (2018). Stage at which riluzole treatment prolongs survival in patients with amyotrophic lateral sclerosis: a retrospective analysis of data from a dose-ranging study. Lancet Neurol.

[5] Hardiman O, van den Berg LH (2017). Edaravone: a new treatment for ALS on the horizon?. Lancet Neurol.

[6] Petrov D, Mansfield C, Moussy A, Hermine O (2017). ALS clinical trials review: 20 years of failure. Are we any closer to registering a new treatment?. Front Aging Neurosci.

[7] Katyal N, Govindarajan R (2017). Shortcomings in the current amyotrophic lateral sclerosis trials and potential solutions for improvement. Front Neurol.

[8] Cedarbaum JM, Stambler N, Malta E, Fuller C, Hilt D, Thurmond B, Nakanishi A (1999). The ALSFRS-R: a revised ALS functional rating scale that incorporates assessments of respiratory function. BDNF ALS Study Group (Phase III). J Neurol Sci.

[9] ClinicalTrials.

[10] Prell T, Ringer T, Witte O, Grosskreutz J (2015). Heart rate variability is decreased in patients with amyotrophic lateral sclerosis. Clin Neurophysiol.

[11] Yunusova Y, Green JR, Greenwood L, Wang J, Pattee GL, Zinman L (2012). Tongue movements and their acoustic consequences in amyotrophic lateral sclerosis. Folia Phoniatr Logop.

[12] Rowland LP, Shneider NA (2001). Amyotrophic lateral sclerosis. N Engl J Med.

[13] Brooks BR, Miller RG, Swash M, Munsat TL, World Federation of Neurology Research Group on Motor Neuron Diseases (2000). El Escorial revisited: revised criteria for the diagnosis of amyotrophic lateral sclerosis. Amyotroph Lateral Scler Other Motor Neuron Disord.

[14] Lomen-Hoerth C, Anderson T, Miller B (2002). The overlap of amyotrophic lateral sclerosis and frontotemporal dementia. Neurology.

[15] Fang T, Jozsa F, Al-Chalabi A (2017). Nonmotor symptoms in amyotrophic lateral sclerosis: a systematic review. Int Rev Neurobiol.

[16] Chida K, Sakamaki S, Takasu T (1989). Alteration in autonomic function and cardiovascular regulation in amyotrophic lateral sclerosis. J Neurol.

[17] Hashimoto T, Yoshiuchi K, Inada S, Shirakura K, Wada N, Takeuchi K, Matsushita M (2015). Physical activity of elderly patients with rheumatoid arthritis and healthy individuals: an actigraphy study. Biopsychosoc Med.

[18] van Buul AR, Kasteleyn MJ, Chavannes NH, Taube C (2018). Physical activity in the morning and afternoon is lower in patients with chronic obstructive pulmonary disease with morning symptoms. Respir Res.

[19] Lipsmeier F, Taylor KI, Kilchenmann T, Wolf D, Scotland A, Schjodt-Eriksen J, Cheng W, Fernandez-Garcia I, Siebourg-Polster J, Jin L, Soto J, Verselis L, Boess F, Koller M, Grundman M, Monsch AU, Postuma RB, Ghosh A, Kremer T, Czech C, Gossens C, Lindemann M (2018). Evaluation of smartphone-based testing to generate exploratory outcome measures in a phase 1 Parkinson's disease clinical trial. Mov Disord.

[20] Linden D, Diehl RR, Berlit P (1998). Reduced baroreflex sensitivity and cardiorespiratory transfer in amyotrophic lateral sclerosis. Electroencephalogr Clin Neurophysiol.

[21] Pinto S, Pinto A, De Carvalho M (2012). Decreased heart rate variability predicts death in amyotrophic lateral sclerosis. Muscle Nerve.

[22] Lundy DS, Roy S, Xue JW, Casiano RR, Jassir D (2004). Spastic/spasmodic vs tremulous vocal quality: motor speech profile analysis. J Voice.

[23] Rong P, Yunusova Y, Wang J, Zinman L, Pattee GL, Berry JD, Perry B, Green JR (2016). Predicting speech intelligibility decline in amyotrophic lateral sclerosis based on the deterioration of individual speech subsystems. PLoS One.

[24] Ellis RJ, Zhu B, Koenig J, Thayer JF, Wang Y (2015). A careful look at ECG sampling frequency and R-peak interpolation on short-term measures of heart rate variability. Physiol Meas.

[25] Task Force of the European Society of Cardiology and the North American Society of Pacing Electrophysiology (1996). Heart rate variability: standards of measurement, physiological interpretation and clinical use. Circulation.

[26] Andreu-Perez J, Garcia-Gancedo L, McKinnell J, van der Drift A, Powell A, Hamy V, Keller T, Yang G (2017). Developing fine-grained actigraphies for rheumatoid arthritis patients from a single accelerometer using machine learning. Sensors (Basel).

[27] (2017). Clinical Trials Transformation Initiative.

[28] Kuss O, Schumann B, Kluttig A, Greiser KH, Haerting J (2008). Time domain parameters can be estimated with less statistical error than frequency domain parameters in the analysis of heart rate variability. J Electrocardiol.

[29] Yunusova Y, Graham NL, Shellikeri S, Phuong K, Kulkarni M, Rochon E, Tang-Wai DF, Chow TW, Black SE, Zinman LH, Green JR (2016). Profiling Speech and Pausing in Amyotrophic Lateral Sclerosis (ALS) and Frontotemporal Dementia (FTD). PLoS One.

[30] WebCite.

[31] DasMahapatra P, Chiauzzi E, Bhalerao R, Rhodes J (2018). Free-living physical activity monitoring in adult US patients with multiple sclerosis using a consumer wearable device. Digit Biomark.

[32] Crouthamel M, Quattrocchi E, Watts S, Wang S, Berry P, Garcia-Gancedo L, Hamy V, Williams RE (2018). Using a ResearchKit Smartphone App to Collect Rheumatoid Arthritis Symptoms From Real-World Participants: Feasibility Study. JMIR Mhealth Uhealth.

[33] Gimeno-Santos E, Raste Y, Demeyer H, Louvaris Z, de Jong C, Rabinovich RA, Hopkinson NS, Polkey MI, Vogiatzis I, Tabberer M, Dobbels F, Ivanoff N, de Boer WI, van der Molen T, Kulich K, Serra I, Basagaña X, Troosters T, Puhan MA, Karlsson N, Garcia-Aymerich J, PROactive consortium (2015). The PROactive instruments to measure physical activity in patients with chronic obstructive pulmonary disease. Eur Respir J.

[34] Andres PL, Skerry LM, Munsat TL, Thornell BJ, Szymonifka J, Schoenfeld DA, Cudkowicz ME (2012). Validation of a new strength measurement device for amyotrophic lateral sclerosis clinical trials. Muscle Nerve.

[35] Doherty A, Jackson D, Hammerla N, Plötz T, Olivier P, Granat MH, White T, van Hees VT, Trenell MI, Owen CG, Preece SJ, Gillions R, Sheard S, Peakman T, Brage S, Wareham NJ (2017). Large scale population assessment of physical activity using wrist worn accelerometers: The UK Biobank study. PLoS One.

[36] Bourdillon N, Schmitt L, Yazdani S, Vesin JM, Millet GP (2017). Minimal window duration for accurate HRV recording in athletes. Front Neurosci.

[37] Bot BM, Suver C, Neto EC, Kellen M, Klein A, Bare C, Doerr M, Pratap A, Wilbanks J, Dorsey ER, Friend SH, Trister AD (2016). The mPower study, Parkinson disease mobile data collected using ResearchKit. Sci Data.

