# Supplementary material for: Objectively Monitoring Amyotrophic Lateral Sclerosis Patient Symptoms During Clinical Trials With Sensors: Observational Study
Source: JMIR Mhealth Uhealth. 2019 Dec 20;7(12):e13433. doi: 10.2196/13433 (PMC6942190; doi:10.2196/13433)
Supplement: Multimedia Appendix 1 [file mhealth_v7i12e13433_app1.docx]

**Supplementary Materials**

**Methods**

*Physical Activity Algorithms*

The activity score is used as a measure of physical activity and is based on the intensity of movement. A frequency of 0.6–5 Hz is typical of human movement. Hence the activity score was computed by integrating the power spectral density of the magnitude of the acceleration vector in the frequency range of 1–10 Hz. Higher frequency movements such as running will produce peak frequencies with a greater power, resulting in a higher activity score. The activity score was computed on windows of 60 seconds. ‘Total activity scores’ (normalized to a per-hour unit) were calculated as the sum of the activity scores calculated for each 60 second window; ‘Maximum Activity Scores’ were the maximum activity scores (per hour) for the monitoring period, and ‘Mean Maximum Activity Scores’ were derived as the mean of the daily maximum activity scores (per-hour) for each day of the monitoring periods.

During the Pilot Study Phase, physical activity algorithms were developed to classify ‘walking’, ‘going up/downstairs’, ‘lying’ and ‘sedentary but not lying’. ‘Lying’ and ‘sedentary but not lying’ classification algorithms showed 100% sensitivity, 100% specificity and 1.0 accuracy when tested against the reference tasks performed by 4 patients at the clinical site (clinic reference task data was available for 4 of the 5 patients at the Pilot Study Phase). The validation of algorithms to classify ‘walking’ and ‘going up/downstairs’ was not as successful, with sensitivity 93% for ‘walking’ and 41% for ‘going up/downstairs’. Algorithm performance was prioritized and therefore the acceleration data were re-examined using a combined category for ‘walking’ and ‘going up/down stairs’ denoted as ‘active’. With this new activity classifier, all 3 states ‘lying’, ‘sedentary but not lying’ and ‘active’ demonstrated 100% sensitivity, 100% specificity and accuracy of 1.0. Future work may investigate whether endpoints based on ‘walking’ and ‘going up/downstairs’ categories may offer advantages to evaluate disease progression over endpoints based on a combined ‘active’ category.

Hence the activity classification algorithm carried over to the Core Study Phase distinguished between ‘active’, ‘lying’ and ‘sedentary but not lying’ classes. Splitting between ‘active’ and ‘sedentary’ classes (‘lying’ and ‘sedentary but not lying’) was based on a measure of physical activity with an appropriate cut point. An additional feature (tilt angle) was required to further split ‘sedentary’ into ‘lying’ or ‘sedentary but not lying’.

The approach lends itself to a simple decision tree as illustrated in Supplementary Figure 1. The algorithm compared the log activity score with a cut-point: if the activity score was above the cut-point the data was classified as ‘active’, if the log activity score was below the cut-point, the data was classified as ‘sedentary’. If the data was ‘sedentary’, the average sensor tilt was computed where the tilt angle from vertical was calculated as

$$tilt= \frac{\pi}{2}-\left| \tan^{-1} \left( \frac{gX}{\sqrt{{gY}^{2}+{gZ}^{2}}} \right) \right|$$

If the sensor tilt was below the threshold, the data was classified as ‘sedentary but not lying’ else the data was classified as lying.

**
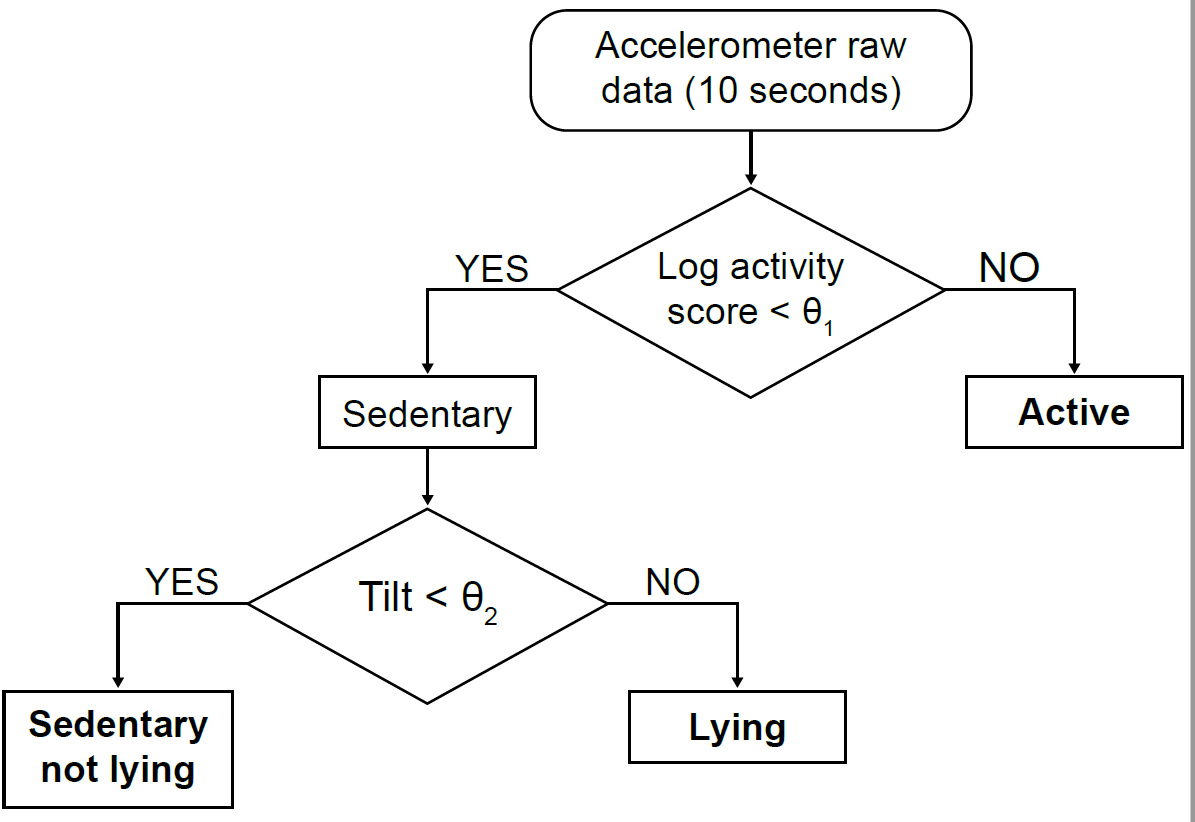
**

**Supplementary Figure 1.** Decision tree for the three-class classifier.

The cut-point values *θ*_1_ and *θ*_2_ were found using the Classification and Regression Trees algorithm known as CART. This approach was validated using a leave-one-patient-out validation procedure using data collected from 29 healthy volunteers (i.e. a ground truth). This evaluated the performance of the algorithm when trained on all but one volunteer (leaving one patient out). The performance of the algorithm was subsequently tested using the data from the one patient not used in the training set. This was repeated across all the volunteers to evaluate how well the algorithm generalized to different training data. Finally, the model was tested using the independent patient dataset collected during the study clinic visits. A comparison between the training (healthy volunteers) and testing data (patients with ALS) showed excellent algorithm performance on both patients and healthy volunteers. Supplementary Figure 2 illustrates the algorithm development and validation process.


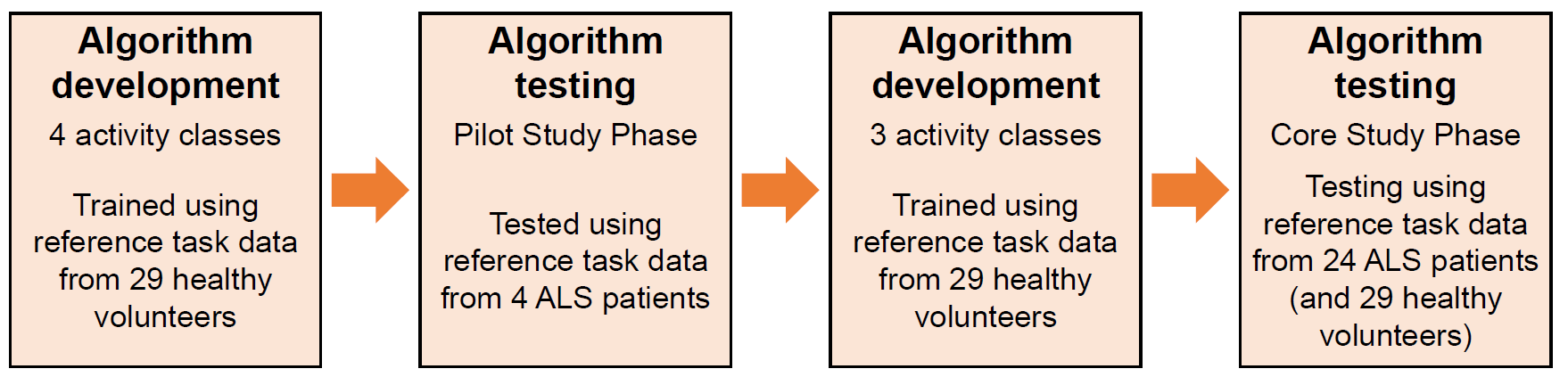


**Supplementary Figure 2.** Algorithm development and validation process.

*Heart-rate Variability Algorithms*

Before computing the RMSSD and LF/HF metrics, several pre-processing steps were followed. These steps were original (although informed by prior work in heart rate variability analysis software, e.g. [1] [2]) and are summarized in Supplementary Table 1:

| **Pre-processing steps** | **RMSSD** | **LF/HF** |
| --- | --- | --- |
| Remove any values that are indicative of non-wear or poor skin contact | Yes | Yes |
| Outlier detection based on the difference between the raw and filtered data | Yes | Yes |
| Replace outliers with the mean of the 8 closest data points | Yes | Yes |
| Detrending (using a linear least square fit to the tRR) | No | Yes |

**Supplementary Table 1.** Summary of inter-beat pre-processing steps

Outlier detection was determined if the difference between the raw tRR and the filtered tRR data was greater than a threshold; detecting large residuals between a model (in this case a low pass filter) and the raw data is a common time series anomaly detection technique, see for example [2]). More concretely, an outlier was detected if

$$s- \tilde{s}>0.03 (ms)$$

In this equation

$$s=\left| rr-\tilde{rr} \right|$$

and $\tilde{rr}$ is the filtered tRR using a zero-phase shift first order low pass filter with a normalized cut off frequency of 0.3 Hz. Here $\tilde{s}$ is the mean of $s.$ The parameter 0.03 is tuneable and was selected based on internal test cases.

Additionally, several data quality checks were performed. These are included in Supplementary Table 2. If any of these checks failed, the window of tRR data was not used in the analysis.

| **Quality check** | **RMSSD** | **LH/HF** |
| --- | --- | --- |
| Time stamps are in increasing order | Yes | Yes |
| The difference between time stamps and tRR are equal (within tolerance limits) | Yes | Yes |
| The derived heart rate is within human limits  Min = 35 bpm, max = 220 bpm | Yes | Yes |
| Sensor is worn correctly - limited variation in heart rate (RMSSD <10 indicates a poor connection or improper use) | Yes | Yes |
| Not too many consecutive outliers - indicate if there are more than 6 consecutive outliers since this may affect reliability of outlier replacement | Yes | Yes |
| Maximum amount of data missing or removed due to poor quality (details below) | Yes, set at 12% | Yes, set at 1% |

**Supplementary Table 2.** Summary of tRR quality checks

For each 5-minute window of tRR data (sliding window with no overlap) , several data points may be missing or removed if they are poor quality or outside reasonable limits. In these cases, it was still possible to compute both the RMSSD and LF/HF metrics, but the reliability of the derived metric was reduced. To better understand the sensitivity of each metric with respect to missing data, a simple sensitivity analysis was performed. A 5% change or greater from the baseline was considered a significant change and the computed values regarded as unreliable. This analysis was used to determine 12% and 1% thresholds in Supplementary Table 2.

From this analysis, the RMSSD metric is less sensitive to the amount of data removed. Around 88% of the data was needed to reliably compute RMSSD for a 5-minute window whilst almost all the data (99%) was needed to reliably compute LF/HF. Hence the RMSSD may be considered a metric that is more robust for patients with lower data quality.

*Speech Algorithms*

In contrast to physical activity and HRV, the data and processing of speech was done offline using a MATLAB script to analyze the speech data and generate a CSV file with the desired endpoints. Mathematical algorithms are based on existing speech processing techniques and used, where possible, open source code that was adapted for optimum implementation.

A summary of the speech processing is shown in Supplementary Figure 3.


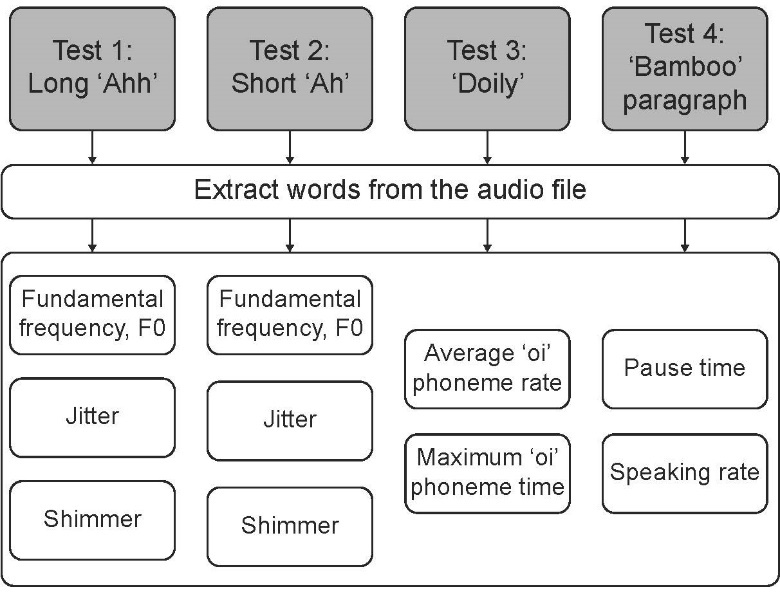


**Supplementary Figure 3.** The speech processing flow.

Firstly, the algorithms detected words within the audio file. A spectral subtraction technique based on Boll [3] was used to detect the start and endpoints of spoken segments; a MATLAB implementation can be found on the Mathworks File Exchange (http://uk.mathworks.com/matlabcentral/fileexchange/7675-boll-spectral-subtraction). Furthermore, to detect the beginning and end of spoken voice, the analysis was conducted on a frame-by-frame basis using an implementation of the “Endpoint Detector”, also available on the Mathworks File Exchange (https://uk.mathworks.com/matlabcentral/fileexchange/45314-endpoint-detector). The frame lengths have been selected experimentally for each of the speech tests. The endpoints were computed as follows:

The fundamental frequency, ‘F0’ was calculated using the Nearly Defect Free (NDF) algorithm [4]. This used both time domain and frequency domain information to provide a smooth estimation of the fundamental frequency and periodicity information. The periodicity output from this algorithm was also used for the calculation of jitter and shimmer.

Jitter – the cycle-to-cycle variation of the fundamental frequency – was calculated from the fundamental frequency vector. The values computed were given as a percentage by calculating the mean of the difference of output from the NDF algorithm.

Shimmer – the cycle-to-cycle variations of amplitude – was calculated from the speech in
25 ms windows with a 75% overlap. The number computed as the endpoint value was the relative shimmer over the windows as a percentage.

The F2 formant was extracted from the word ‘doily’ using a formant tracker algorithm [5] which also enabled further analysis of the F2 characteristics. Maximum phonation time was computed as the maximum detected duration of the ‘oi’ phoneme across the three repeats of the word ‘doily’. Average phoneme rate was extracted from the F2 slope.

The pause time was computed as a percentage of the time taken to speak the first-to-last detected words minus the time spent in talking. The speaking rate assumed all 99 words were spoken and therefore used the time taken to speak the first-to-last detected words to report words per minute.

**References**

[1] Ramshur, J. T. “Design, evaluation, and application of Heart Rate Variability Analysis Software (HRVAS)”, 10.13140/RG.2.2.33667.81444

[2] [Thuraisingham, R. A](https://www.ncbi.nlm.nih.gov/pubmed/?term=Thuraisingham%20RA%5BAuthor%5D&cauthor=true&cauthor_uid=16806571). “Preprocessing RR interval time series for heart rate variability analysis and estimates of standard deviation of RR intervals”, [Computer Methods and Programs in Biomedicine](https://www.sciencedirect.com/science/journal/01692607), Vol. 83 (1), 78, 2006.

[3] Boll, S. F. “Suppression of Acoustic Noise in Speech Using Spectral Subtraction”, IEEE Transactions on Acoustics, Speech, and Signal Processing, Vol. 27 (2), 113, 1979.

[4] Kawahara, H, et al. “Nearly defect-free F0 trajectory extraction for expressive speech modifications based on STRAIGHT”, Eurospeech, 9th European Conference on Speech Communication and Technology, 537-540, 2005.

[5] Mustafa, K & Bruce, I. “Robust formant tracking for continuous speech with speaker variability”, IEEE Transactions on Audio, Speech, and Language Processing, Vol. 14 (2), 435-444, 2006.
